# The Nanomechanical Performance and Water Uptake of a Flowable Short Fiber Composite: The Influence of Bulk and Layering Restorative Techniques

**DOI:** 10.3390/polym17111553

**Published:** 2025-06-02

**Authors:** Tamás Tarjányi, András Gábor Jakab, Márton Sámi, Krisztián Bali, Ferenc Rárosi, Maja Laura Jarábik, Gábor Braunitzer, Dániel Palkovics, Lippo Lassila, Edina Lempel, Márk Fráter, Sufyan Garoushi

**Affiliations:** 1 Department of Medical Physics and Informatics, University of Szeged, 6720 Szeged, Hungary; tarxtamas@gmail.com (T.T.); rarosi.ferenc@med.u-szeged.hu (F.R.); 2 SEMILAB Semiconductor Physics Laboratory Co. Ltd., 1117 Budapest, Hungary; krisztian.bali@semilab.hu; 3 Department of Operative and Esthetic Dentistry, Faculty of Dentistry, University of Szeged, 6720 Szeged, Hungary; jakab.andras.gabor@gmail.com (A.G.J.); jarabikmajalaura13@gmail.com (M.L.J.); 4 Department of Optics and Quantum Electronics, University of Szeged, 6720 Szeged, Hungary; samimarton11@gmail.com; 5 dicomLAB Dental Ltd., 6726 Szeged, Hungary; braunitzergabor@gmail.com; 6 Department of Periodontology, Faculty of Dentistry, Semmelweis University, 1085 Budapest, Hungary; dpalkovics@gmail.com; 7 Department of Biomaterials Science and Turku Clinical Biomaterials Center-TCBC, Institute of Dentistry, University of Turku, 20014 Turku, Finland; liplas@utu.fi (L.L.); sufgar@utu.fi (S.G.); 8 Department of Restorative Dentistry and Periodontology, University of Pécs Medical School, 7624 Pécs, Hungary; lempel.edina@pte.hu

**Keywords:** short-fiber-reinforced composite, nanomechanical properties, nanoindentation, bulk-fill technique, creep resistance, water uptake, restorative dentistry

## Abstract

This study aimed to evaluate the nanomechanical surface properties and water uptake of a flowable short-fiber-reinforced composite (SFRC) using various restorative techniques in order to assess its potential as a standalone restorative material. Nanoindentation and compressive creep testing were employed to characterize material performance. Three resin composites were examined: a flowable SFRC (everX Flow), a bulk-fill particulate filler composite (PFC), and a conventional PFC. Five experimental groups were established based on the restorative technique: layered PFC, layered SFRC, bulk SFRC, bulk PFC, and a bi-structure combining SFRC and PFC. Ninety standardized specimens (n = 18/group) were fabricated. Static and creep nanoindentation tests were conducted to assess surface properties, and water uptake was measured over a 30-day period. Data were analyzed using one-way ANOVA and Bonferroni post hoc tests. Nanoindentation revealed significant differences in hardness, with bulk PFC exhibiting the lowest values (*p* < 0.001). Creep testing indicated changes in modulus and viscosity following water storage. Notably, bulk SFRC showed the lowest water absorption (*p* < 0.001). Overall, bulk-applied SFRC demonstrated favorable nanomechanical properties and reduced water uptake, demonstrating its suitability as a standalone restorative material. Further clinical investigations are recommended to validate its long-term performance.

## 1. Introduction

Resin composite restorative materials are extensively used by clinicians to restore lost tooth structure in both anterior and posterior teeth [1,2]. Direct composite restorations offer an efficient, cost-effective, and reliable solution for tooth restoration [3]. For small to medium-sized fillings, particulate filler composite (PFC) restorations provide a dependable option, with annual failure rates ranging from 1 to 3% [4]. However, Bernardo et al. demonstrated a strong correlation between restoration size and survival rates [5]. They reported an annual failure rate of 0.95% for single-surface PFC fillings [5], which increased to over 9.43% for PFC restorations involving four or more surfaces [5]. The most common causes of PFC restoration failures were secondary decay and fractures [5,6,7].

Excessive cavity preparations resulting in high-volume cavities, missing marginal ridges, and root canal treatments increase the susceptibility of teeth to future fractures [8,9]. The greater the loss of dentine due to these factors, the higher the risk of tooth fracture. To address these challenges, modern restorations aim not only to restore the aesthetics and function of teeth, but also to reinforce and protect the remaining tooth structure against fractures [10]. While PFC materials are the most commonly used for direct restorations, their physical limitations can lead to clinical failures, particularly in large or deep cavities. PFC materials face two primary challenges: polymerization shrinkage and limited fracture toughness, despite being brittle yet strong [11,12,13,14,15].

The issue of low fracture toughness becomes particularly significant in larger direct restorations, where the volume of the material increases [15]. Fracture toughness, a critical mechanical property, represents a material’s ability to resist the catastrophic propagation of flaws under applied loads [16,17,18]. This parameter is a key indicator of fatigue resistance, which helps predict the material’s structural performance [19]. PFC materials fall considerably short in fracture toughness compared to that of sound dentine [20]. A potential solution is the use of short-fiber-reinforced composite (SFRC) materials to replace a substantial portion of the missing dentine [21].

In 2013, a packable SFRC material, EverX Posterior, was introduced and recommended for use in high-stress-bearing areas [22,23]. This was followed in 2019 by a flowable version, EverX Flow, which demonstrated improved surface properties compared to the paste-consistency material [24]. The original version contained glass fibers measuring 0.3–1.5 mm in length and 17 µm in diameter, while the flowable version incorporates micro-glass fibers with an average length of 200–300 µm and a diameter of 6 µm. This material shows a high degree of similarity to dentine in both microstructure and physical properties [25,26]. The fracture toughness of EverX Flow is notably higher (2.8 MPa·m^1/2^) than that of conventional PFC materials (1.6 MPa·m^1/2^), approaching the range observed for dentine (2.3–3.5 MPa·m^1/2^) [21].

In general, resin composites encompass a diverse range of formulations, some of which can be applied using either traditional layering techniques, where incremental layers are built up, or bulk-fill techniques, which allow for faster application by filling the cavity in a single layer. Both techniques are commonly used in restorative dentistry, offering clinicians flexibility in treatment based on the cavity size, location, and clinical situation. However, there are still controversial results regarding the preference for each technique, with varying opinions on their efficacy and clinical outcomes [27,28].

Previous studies have shown that even deep mesio-occluso-distal (MOD) cavities can be effectively reinforced using SFRCs in direct restorations [15,29]. Conventionally, SFRC materials have been employed as dentine replacements in direct restorations, typically covered with a layer of PFC in line with manufacturer recommendations [21]. However, research indicates that maximizing the amount of fibers within restorations enhances their reinforcing effect [21,30,31]. This raises the question of whether fiber-reinforced materials could be used without a conventional PFC covering layer. Garoushi et al. demonstrated that large MOD cavities achieved greater reinforcement when restored exclusively with SFRC compared to when SFRC was used as a base beneath a PFC layer [32]. To be used in vivo without a covering layer, the material must withstand the challenging environment of the oral cavity. Earlier fiber-reinforced materials were prone to excessive wear, resulting in a rough plaque-retentive surface [33,34]. However, modern flowable SFRCs have shown significant improvements in this regard, meeting the American Dental Association’s wear criteria [24,35]. Rawda et al. reported satisfactory clinical outcomes in a trial where flowable SFRC was used without proximal surface coverage following an 18-month observation period [36]. For these reasons, SFRCs show promise for future clinical use as complete restorations. However, further questions must be addressed to fully realize this potential.

The aim of this study is to evaluate the nanomechanical surface properties and water uptake of flowable SFRC under different restorative techniques. Using nanoindentation and bulk compressive creep testing, we assessed the material’s suitability for use with or without PFC coverage, offering insights into its potential as a standalone restorative material.

Our first null hypothesis, concerning surface hardness, was that there would be no significant differences among the tested materials. For water degradation and water uptake, our second null hypothesis posited that the investigated SFRC material would not differ significantly from the PFC. Finally, our third null hypothesis was that there would be no significant differences between the research groups (bulk vs. layering) regarding bulk compressive creep.

## 2. Materials and Methods

Details of the materials and information from the manufacturers are listed in Table 1. This study examined three types of resin composites, a flowable bulk-shade SFRC (everX Flow), a bulk-fill PFC composite (SDR flow+), and a conventional PFC composite (G-aenial Posterior), each applied using different layering techniques.

Eighteen standard-size composite specimens were fabricated for each group using a 5 × 5 × 5 mm metallic mold (*n* = 18/group). The specimens were prepared with different materials and layering strategies according to five study groups (Table 2).

Group 1 (control group): three consecutive layers of a conventional packable PFC (G-aenial Posterior) were applied in 2 mm, 2 mm, and 1 mm thick layers, respectively. Each layer was photopolymerized for 20 s according to the manufacturers’ instructions using a high-performance hand-held curing lamp (D-Light Pro, GC Europe, Leuven, Belgium). The average power density of the photopolymerization unit was 940 ± 20.8 mW/cm^2^. This was measured with a digital radiometer (Jetlitelight tester, J. Morita USA Inc. Irvine, CA, USA) before performing the restorations.

Group 2 (layered SFRC): three consecutive layers of a flowable SFRC were applied in the same manner as in Group 1. Each layer was photopolymerized for 20 s.

Group 3 (bulk SFRC): the same flowable SFRC material used in Group 2 was applied in a single 5 mm thick layer (bulk-fill technique). Each specimen was photopolymerized for 20 s.

Group 4 (bulk PFC): a flowable bulk-fill PFC material was used in the same way as described for Group 3. The specimens were photopolymerized for 20 s.

Group 5 (bi-structure): two consecutive layers (2 mm each) of SFRC were applied, with each layer photopolymerized for 20 s. The SFRC was then covered with a 1 mm layer of PFC, as described in Group 1.

After photopolymerization, the specimens were stored under dry conditions at room temperature to allow for post-polymerization curing for several days. Each specimen was then polished using silicon carbide (SiC) abrasive papers with progressively finer grits. Polishing began with P1500 grit until the surface was smooth, followed by P2000 and P2500 grits until surface scratches were eliminated and an appropriate surface roughness was achieved. In total, at least a 100 μm surface layer was removed during the polishing process. After polishing, the specimens were mounted onto a stainless-steel specimen holder using an optical adhesive diluted with acetone. The adhesive mixture was applied to the surface of the specimen holder, and the specimens were pressed continuously until the adhesive dried to ensure proper adhesion.

### 2.1. Nanoindentation Protocol

**Figure 1:**
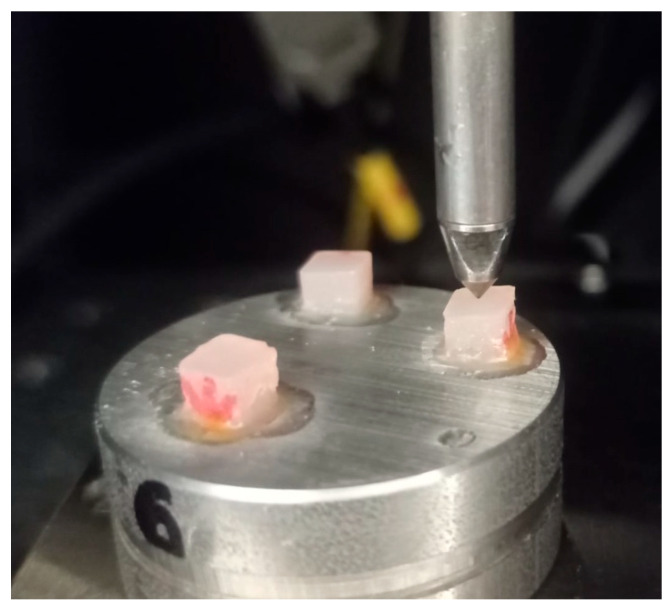
Nanoindentation measurement setup. The composite specimens were polymerized on the top side with the curing lamp positioned accordingly. The arrow on the specimens indicates the direction of polymerization, pointing toward the bottom surface.

The nanomechanical surface properties of the composite specimens were tested using the IND-1500 nanoindenter (Semilab, Budapest, Hungary) one week after the specimens were fabricated and dry-stored at room temperature. A Berkovich diamond tip provided by Semilab was used for the nanoindentation tests (Figure 1). A fresh area correction function for the tip was pre-measured on fused silica, and the compliance of the measurement device was calibrated and set to 0.0003 μm/mN. The measurements and analysis followed the ISO 14577 standard [37]. The device operated in force control mode with a maximum force set at 20 mN, an initial contact force of 0.15 mN, and a hold at maximum force for 1 s. Each specimen underwent 19 nanoindentations at 40 μm intervals between indents. Each group consisted of 18 specimens, which were randomly divided into three subgroups: 6 specimens for topside measurements, 6 for side measurements, and 6 for bottom measurements. This resulted in a total of 1710 nanoindentations (19 indents × 18 specimens × 5 groups) on the dry specimens. The Poisson’s ratio for the composite materials was set at 0.27 for the analysis. The analysis was conducted using the method of Oliver and Pharr [38].

### 2.2. Nanoindentation Creep

Additional creep measurements were conducted on the six specimens from each group following the initial static nanoindentation test. Each specimen underwent 10 creep measurements at 50 μm intervals using the IND-1500 nanoindenter, equipped with a Berkovich diamond tip (50 nm radius of curvature), at room temperature. The loading force for the creep measurements was maintained at a constant 25 mN over a 300 s period, during which 300 data points were collected.

**Figure 2:**
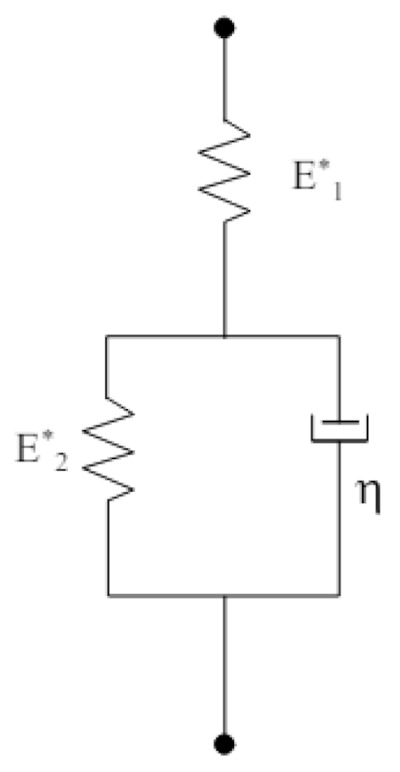
Schematic representation of the standard linear viscoelastic model.

The standard linear model was used to evaluate the creep behavior by fitting the time-dependent penetration curve. This model consists of a Hooke body connected in series to a Kelvin–Voigt model, which comprises a Hooke and Newton body connected in parallel, as illustrated in Figure 2.

The analytical solution for the displacement in the case of constant loading (creep) is represented by the following equation:

$$h^{2}(t)=\frac{\pi }{2}F\cdot \cot\alpha \left[\frac{1}{E_{1}^{*}}+\frac{1}{E_{2}^{*}}\left(1-e^{-\frac{E_{2}^{*}t}{\eta }}\right)\right]$$

where F represents the applied loading force, α is the angle of the conical indenter tip, and $E_{1}^{*}$, $E_{2}^{*}$, and η are the combined modulus of elasticity and viscosity values of the standard linear model. The least squares method was applied to the measured h(t) time-dependent displacement curves to fit the standard linear model. The specimen modulus value can then be calculated from the fitted $E_{1}^{*}$, $E_{2}^{*}$ combined modulus values using the following formula:

$$\frac{1}{E^{*}}=\frac{1-\nu_{s}^{2}}{E_{s}}+\frac{1-\nu_{i}^{2}}{E_{i}}$$

where $E_{s}\;$ is the specimen modulus, $E_{i}\;$ is the indenter tip modulus (diamond $E_{i}=1050$ GPa), $\nu_{s}\;$ is the Poisson’s ratio of the specimen, and $\nu_{i}\;$ is the Poisson’s ratio of the indenter tip (diamond $\nu_{i}=0.07$).

Additionally, the total displacement during the creep measurements was calculated by recording the displacement at 300 s and subtracting the depth at the start of the measurement. This value is referred to as creep depth in the following sections.

### 2.3. Water Degradation and Uptake

After the initial set of nanoindentation tests, the specimens were removed, and their dimensions and weight were measured using a micrometer screw and an analytical balance (Ohaus Pa224c, with an accuracy of 0.1 mg). The nanoindentation creep test was conducted both before and after a 30-day water-aging period at a laboratory room temperature of 24 °C. The static nanoindentation measurements were repeated on the six top specimens following the water-aging period. The results from the two tests were compared statistically to assess the effect of water degradation.

The dry weight of each specimen was recorded immediately after preparation. During the water-aging period, weights were measured at 1, 2, 3, 4, 10, 14, 28, and 30 days (*m_w_*) using the analytical balance. The absorbed water mass per unit volume uptake was calculated using the following formula:

$$\varphi =\frac{m_{w}-m_{0}}{V},$$

where *m* is the mass measured on the actual day, *m*_0_ is the initial mass at day 0, and *V* is the volume of the composite specimen.

### 2.4. Surface Morphology and Characterization

Scanning electron microscope (SEM) secondary electron images were taken using a Hitachi S-4700 (Hitachi High-Technologies Corporation, Tokio, Japan) field emission cathode scanning electron microscope (FESEM). The surfaces of the specimens were coated with a few nm thin electrically conducting golden layer for the elimination of surface charging. To facilitate the identification of the imprints, extra nanoindentations were performed at a greater loading force to find the imprints.

### 2.5. Statistical Analysis

Statistical analysis was performed using IBM SPSS Statistics, version 23 (IBM Corp., Armonk, NY, USA). The normality of the data was assessed using the Shapiro–Wilk test, and the homogeneity of variances was evaluated with Levene’s test. For group comparisons, a one-way analysis of variance (ANOVA) was conducted, followed by Bonferroni’s post hoc test to identify significant differences between individual groups. Mean values and standard deviations were calculated and reported for each group. A significance level of *p* < 0.05 was considered statistically significant.

Additionally, the creep behavior of the materials was analyzed using a curve fitting approach based on the least squares method to model the time-dependent deformation accurately. This allowed for the extraction of parameters that describe the viscoelastic response over time. All analyses were performed with a minimum of 6 specimens per group to ensure sufficient statistical power.

## 3. Results

### 3.1. Static Nanoindentation

**Figure 3:**
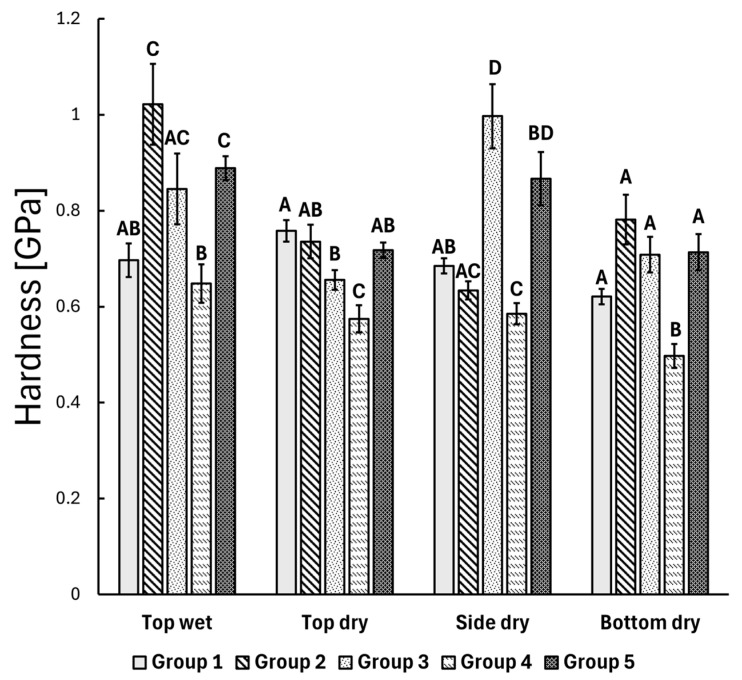
The mean hardness of each composite group measured by the static nanoindentation method. The error bar shows the standard error of the mean. Identical alphabetic letters (A–D) indicate no significant difference, while different letters denote a significant difference.

The prescribed static nanoindentation measurements were conducted on the top and bottom layers of the composite specimens. Additionally, the top surface of the composite blocks was measured both before (dry) and after water storage (wet). The ANOVA revealed significant differences in the mean hardness values between the composite groups (illustrated in Figure 3). In the top layer before water storage, the bulk PFC group (Group 4) exhibited a statistically significantly lower mean hardness value compared to the other groups (*p* < 0.05), and the control group (layered PFC, Group 1) also showed a difference compared to the bulk SFRC specimens (Group 3) (*p* = 0.028). After water storage, the top layer was remeasured on the same specimens, and the Bonferroni post hoc results indicated that a significant difference remained for the bulk PFC group (Group 4) compared to the other groups (*p* < 0.05), except for the control group (layered PFC, Group 1). However, the control group (layered PFC, Group 1) showed a significant difference in mean hardness value compared to the layered SFRC group (Group 2) (*p* < 0.001) and the bi-structure group (Group 5) (*p* < 0.001) after water storage. For the bottom layer, a similar trend was observed as in the top layer before water storage, with the post hoc results showing that the bulk PFC group (Group 4) had a statistically significantly lower mean hardness value compared to the other groups (*p* < 0.05).

### 3.2. Creep Nanoindentation

**Figure 4:**
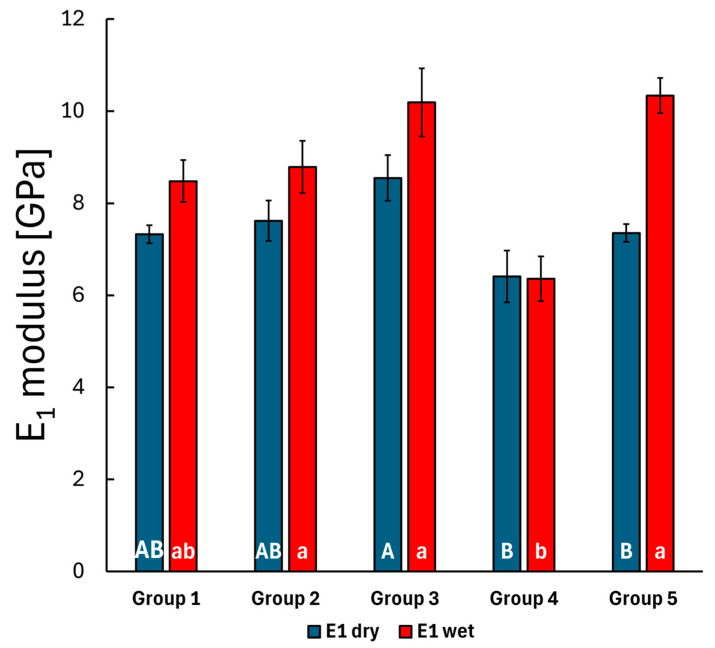
The mean *E_1_*-fitted parameter of the measured creep curves for the composite groups before and after water storage, with error bars representing the standard error of the mean. Lowercase alphabetic letters (a,b) refer to the dry groups; uppercase alphabetic letters (A,B) refer to the wet groups. Identical letters (regardless of case) indicate no significant difference, whereas different letters (irrespective of case) denote a significant difference.

**Figure 5:**
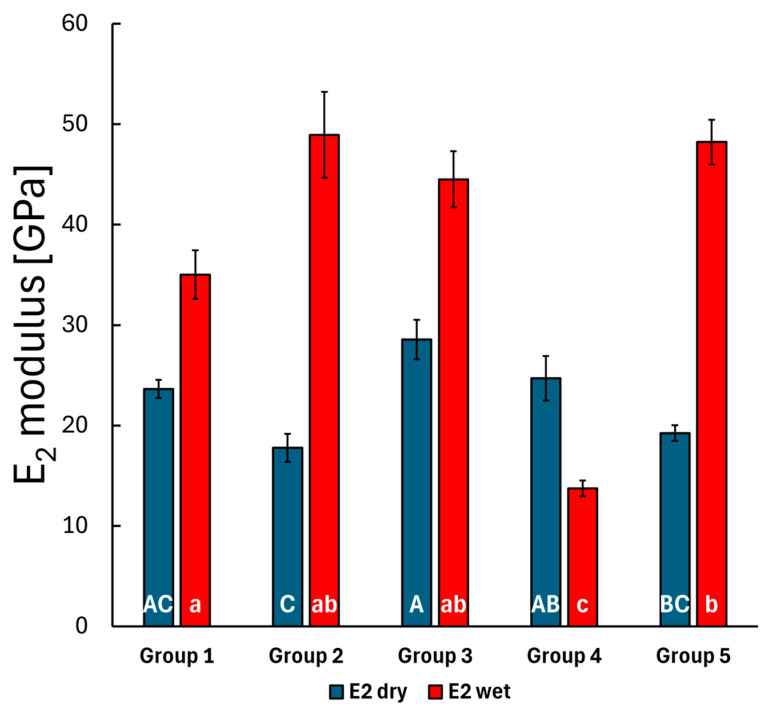
The mean *E_2_*-fitted parameter of the measured creep curves for the composite groups before and after water storage, with error bars representing the standard error of the mean. Lowercase alphabetic letters (a–c) refer to the dry groups; uppercase alphabetic letters (A–C) refer to the wet groups. Identical letters (regardless of case) indicate no significant difference, whereas different letters (irrespective of case) denote a significant difference.

**Figure 6:**
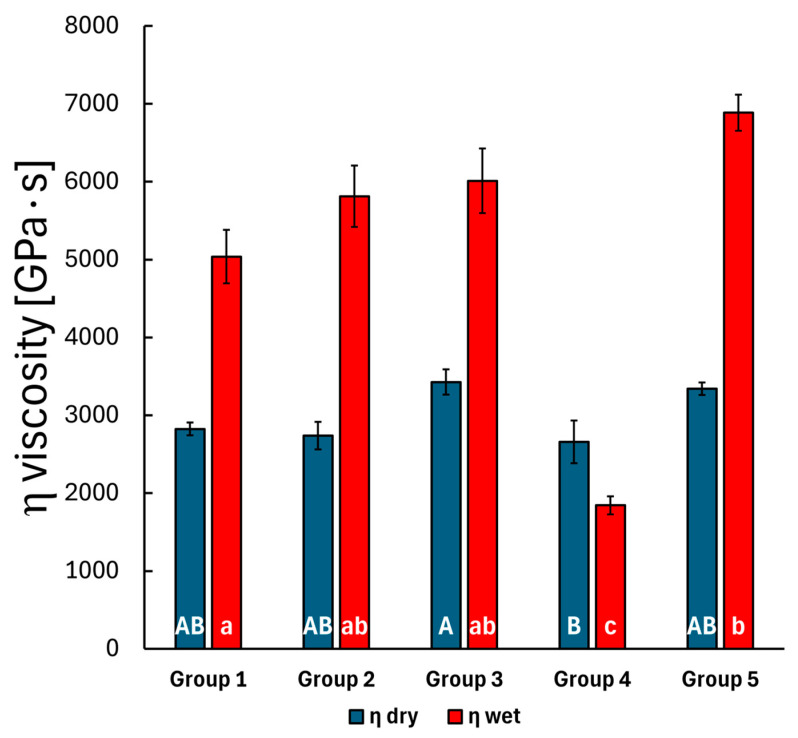
The mean *η*-fitted parameter of the measured creep curves for the composite groups before and after water treatment, with error bars representing the standard error of the mean. Lowercase alphabetic letters (a–c) refer to the dry groups; uppercase alphabetic letters (A–C) refer to the wet groups. Identical letters (regardless of case) indicate no significant difference, whereas different letters (irrespective of case) denote a significant difference.

During the creep measurements, the penetration depth (displacement) was continuously recorded over 300 s under a fixed 10 mN load. The standard linear model, as outlined in the methods, was used to fit the displacement curve with three parameters: *E_1_*, *E_2_* and *η*. The *E_1_* modulus describes the initial elastic behavior at the first measured time point, which relates to the stiffness of the material, while the *E_2_* and *η* parameters characterize the time-dependent behavior of the material, indicating a delayed or retarded response. Measurements were conducted on the top layer of each group, both before and after storing the specimens in distilled water. The results are shown in Figure 4, Figure 5 and Figure 6.

The Bonferroni post hoc test results indicated a significant difference in the mean *E_1_* modulus value between the bulk SFRC specimens (Group 3) and the bulk PFC specimens (Group 4) (*p* = 0.005), while no significant difference was observed for this parameter among the other groups. This slightly changes after storing the specimens in water, as the mean *E_1_* modulus differs significantly in the case of the bulk PFC (Group 4) to the layered SFRC (Group 2) (*p* = 0.031) specimens and to the bulk SFRC specimens (Group 3) (*p* < 0.001). The *E_2_* and *η* parameters showed significant changes after the 30-day water treatment. Before water treatment, the *E_2_* parameter exhibited significant differences between the layered SFRC (Group 2) and the bulk SFRC (Group 3) group (*p* < 0.001), between the layered SFRC (Group 2) and the bulk PFC (Group 4) group (*p* = 0.011), and also between the bulk SFRC (Group 3) and the bi-structure (Group 5) group (*p* = 0.001). The mentioned difference between the layered SFRC (Group 2) and the bulk PFC (Group 4) specimens remained consistent after water treatment (*p* < 0.001), and the bulk SFRC (Group 3) versus the bulk PFC (Group 4) specimens also showed a significant difference (*p* < 0.001). The viscosity parameter for the pre-treated specimens showed a significant difference between the bulk SFRC (Group 3) and the bulk PFC (Group 4) specimens according to the Bonferroni post hoc test (*p* = 0.035). Post water storage, the time-dependent behavior described by the viscosity parameter varied significantly, with the bulk PFC (Group 4) group differing from all other groups (*p* < 0.05), and the control group (layered PFC, Group 1) also differed from the bi-structure group (Group 5) (*p* < 0.001). The mean modulus and viscosity parameters were compared before and after the 30-day water-aging period using a *t*-test. The *E_1_* modulus showed a significant difference after the water treatment in the case of the control group (Group 1, *p* = 0.009) and the bi-structure group (Group 5, *p* < 0.001). The *E_2_* modulus and *η* viscosity parameters showed a significant difference across all groups after the water treatment. Overall, water treatment significantly affected the time-dependent behavior, with notable differences in the retarded modulus and viscosity values.

**Figure 7:**
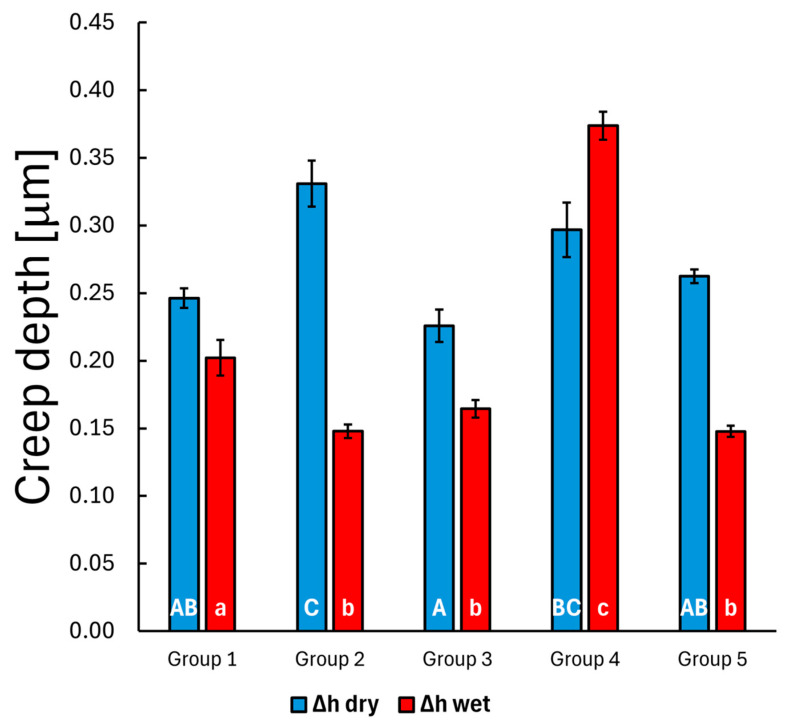
The mean creep depth at 300 s before and after water treatment. The error bars show the standard error of the mean. Lowercase alphabetic letters (a–c) refer to the dry groups; uppercase alphabetic letters (A–C) refer to the wet groups. Identical letters (regardless of case) indicate no significant difference, whereas different letters (irrespective of case) denote a significant difference.

The total penetration depth was recorded during the 300 s creep measurement, and the absolute depth penetration (creep depth, see Figure 7) was calculated by subtracting the initial penetration (displacement at time 0, the initial contact) from the actual measured depth. A statistical test was performed to determine differences in the mean creep depth among the composite groups. The dry specimens generally exhibited higher creep behavior—indicating how much the nanoindenter tip penetrated further into the material after initial contact—except for the bulk PFC group (Group 4), which showed a 0.30 ± 0.02 µm versus 0.37 ± 0.01 µm additional creep penetration at 300 s compared to the initial penetration (see Figure 4). The calculated creep depths were also compared using the Bonferroni post hoc test. In the dry specimens, the control group (layered PFC, Group 1) showed a significant difference compared to the layered SFRC (Group 2) specimens (*p* < 0.001), while the layered SFRC (Group 2) also differed significantly from the bulk SFRC (Group 3) (*p* < 0.001) and the bi-structure (Group 5) specimens (*p* = 0.024); furthermore, the bulk SFRC (Group 3) specimens showed a significant difference compared to the bulk PFC specimens (Group 4) (*p* = 0.042). After 30 days of water storage, the control group (layered PFC, Group 1) showed a significant difference compared to all other groups (*p* < 0.05), and the bulk PFC (Group 4) had a significantly greater mean creep depth than the other groups (*p* < 0.05).

### 3.3. Water Uptake

**Figure 8:**
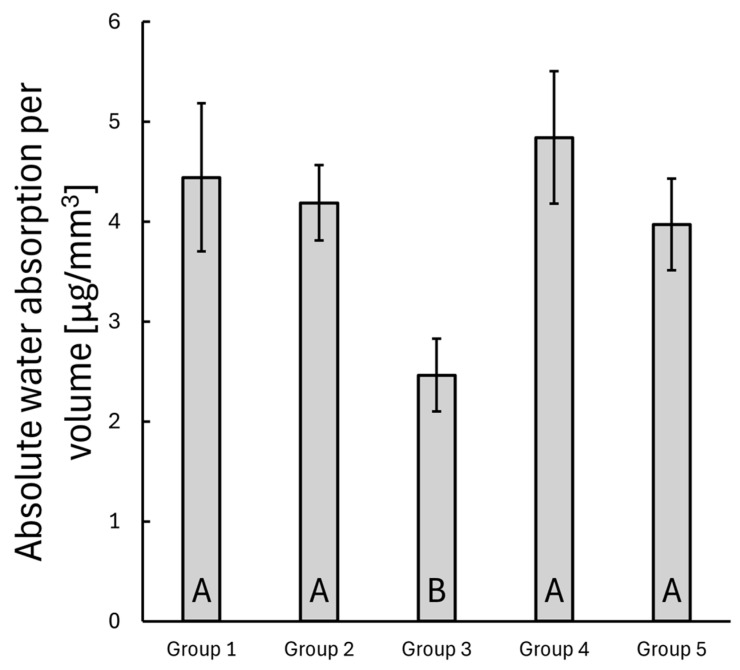
The mean water absorption per unit volume for each composite group at day 30. The error bars represent the standard error of the mean. Different alphabetic letters indicate statistical significance.

The absorbed water mass per unit volume was compared between the groups on day 30. The bulk SFRC specimens (Group 3) exhibited a significant difference compared to all other groups (*p* < 0.05), while the remaining groups showed no statistically significant differences (see Figure 8).

**Figure 9:**
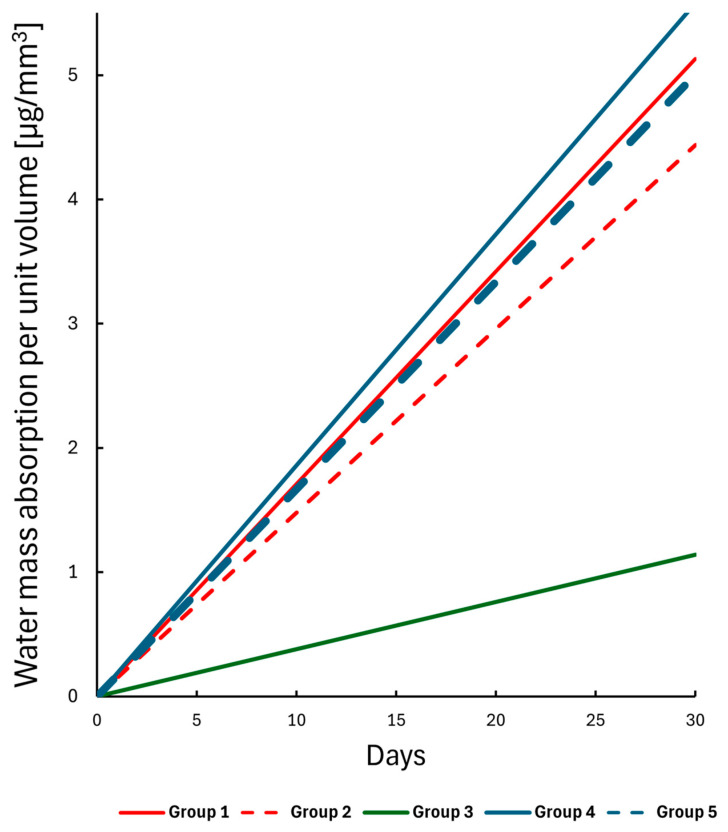
Linear regression lines for the water mass absorption per unit volume dataset over a 30-day interval.

A linear regression analysis was performed on the absorbed water mass per unit volume dataset, revealing a clear linear trend in water absorption over time. The fitted lines are shown in Figure 9. All groups demonstrated a similar water uptake pattern except for the bulk SFRC (Group 3). A strong significant correlation was observed, with slopes indicating a daily uptake range between 0.148 and 0.186 µg/mm^3^, except for the bulk SFRC group (Group 3), where the correlation was weak and the slope was only 0.038 µg/mm^3^ per day.

### 3.4. SEM Evaluation

**Figure 10:**
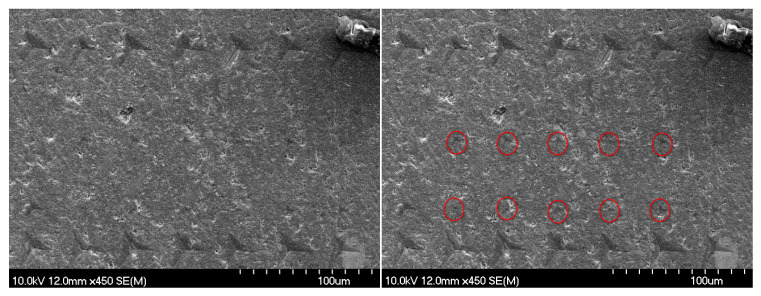

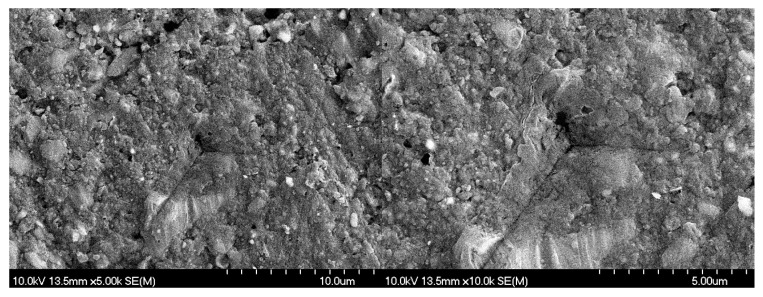
Scanning electron microscope images of the composite (SDR) specimen surface after nanoindentation at different magnifications. The impressions used in the study are marked in red.

In Figure 10, the red-marked indentation imprints correspond to the nanoindentation experiments of this study. The dental composite surface exhibits noticeable inhomogeneity at the nanoscale, which partially describes the observed deviations in the mechanical parameters such as hardness and modulus. Notably, no crack propagations were observed in the SEM images, suggesting that the material can accommodate localized deformation without failure (indicting a high fracture toughness).

## 4. Discussion

Restoring high-C-factor deep cavities remains a significant challenge for clinicians [39,40,41]. This difficulty arises partly from the material-related properties of resin composites, including polymerization shrinkage and insufficient fracture toughness [21,42], and partly from cavity-related factors, such as the C-factor and volume factor [43]. The C-factor is a reliable indicator of shrinkage-induced stress when the cavity volume is comparable [42]. Furthermore, an increase in cavity volume results in greater polymerization shrinkage and more pronounced gap formation [44]. In this study, the dimensions of deep cavity were simulated to reflect their clinical relevance.

The challenges mentioned above are traditionally addressed using an incremental restorative technique. This involves the application of 2 mm thick layers in either an oblique or horizontal direction, with the aim of reducing polymerization-shrinkage-induced stress by modifying the cavity configuration layer by layer, thereby also influencing the C-factor [45]. However, this method is complex, demands considerable chair time, and may still result in the entrapment of voids between layers. As a result, the demand for a simplified technique has led to the development of bulk-fill composites [46]. Bulk-fill materials possess an enhanced depth of cure, achieved either by increasing translucency to facilitate greater light transmission [47] or by utilizing more reactive photoinitiators, stress-relieving monomers, and innovative fillers such as pre-polymer particles and fiberglass rod segments [46]. Nevertheless, the question remains as to whether bulk-fill resin composites should be applied in a single bulk layer or incrementally, and whether they should be capped or not [48,49,50,51].

In our study, SFRC was utilized using multiple direct restorative techniques. The successful application of dental materials as load-bearing structural components in restored teeth requires adequate mechanical properties. Therefore, the general mechanical characterization of candidate materials is essential. A useful starting point is the measurement of stress–strain (or load–deformation) properties [52]. Nanoindentation enables the investigation of selected material properties using small amounts of material based on the load–displacement data of indentations on a submicron scale and has been suggested as being advantageous over conventional methods due to its high resolution of force and precise indent positioning [53,54,55].

In the first part of this study, static nanoindentation was used to evaluate the hardness of different direct restorations at three levels: top, side, and bottom. The bulk PFC (SDR, Group 4) demonstrated significantly lower hardness values at all three levels compared to the other groups (*p* < 0.05). These findings align with previous studies in the literature, where bulk PFC (Group 4) exhibited one of the lowest surface hardness values among the tested resin composites [56,57]. This difference could be attributed to the lower filler content of SDR 68 wt.% (Table 1) and possibly to the lower degree of monomer conversion and cross-linking compared to the other tested resin composites [24,40]. On the other hand, SFRC with a 70 wt.% filler loading exhibited comparable or even higher hardness values at the side and bottom levels compared to the control PFC composite group with a 77 wt.% filler loading. This can be attributed to the unique structure of flowable SFRC, which contains a high proportion of E-glass fibers (25 wt.%) in addition to barium glass particulate filler (45 wt.%). The E-glass fibers, being harder than barium glass particles, likely contribute to the increased hardness observed in the SFRC material. Moreover, the flowable SFRC (bulk shade) is translucent, and its fibers scatter light, which may improve the degree of conversion and enable its use in increments up to 5.5 mm thick. These findings align with those of Lassila et al., who reported that flowable SFRC demonstrated superior performance in all tested mechanical parameters compared to SDR and some conventional PFCs [24].

Upon examining the bottom part of the specimens, the bulk PFC specimen (Group 4) exhibited significantly lower hardness values compared to SFRC groups (*p* < 0.001). This finding is consistent with the results of Karacolak et al., who reported that SDR demonstrated one of the lowest microhardness values at the bottom of the specimens when compared to other bulk-fill materials [58]. Numerous studies have shown a gradual decrease in microhardness values from the “top” toward the “bottom” of both conventional and bulk-fill composite materials, with the extent of this decrease varying significantly depending on the type of resin composite [59,60]. Interestingly, no statistically significant difference in hardness at the bottom of the specimens was observed among the other groups. This indicates that flowable SFRC, whether applied in a layered or bulk manner, produced comparable hardness values. These findings align with those of Fráter et al., who reported no differences in microhardness values, as measured by nanoindentation, between bulk and layered SFRC specimens in artificial root canals [61]. Similarly, our results are consistent with those of Néma et al., who found no differences in the degree of conversion at different depths (top, middle, bottom) between layered and bulk-fill SFRC restorations [42]. This can likely be attributed to the transparent nature of the material and the light transmission facilitated by the short glass fibers [22,62,63,64]. Fráter et al. also demonstrated that there is no difference in fracture resistance between flowable SFRC restorations applied in a layered or bulk manner [65]. Furthermore, Néma et al. emphasized that there is no difference in polymerization-induced crack formation between flowable SFRC restorations applied in either a layered or bulk manner [66]. However, Néma and colleagues also observed that bulk application resulted in less polymerization-induced gap formation compared to the layered application of SFRC material [42].

Interestingly, when examining the specimens after water storage, no difference in hardness was observed between the control group (layered PFC, Group 1) and the bulk PFC (Group 4) specimens. Water has the ability to reduce the surface hardness of restorative materials by acting as a plasticizing molecule within the composite matrix. This process softens the polymer resin component by swelling the network and reducing the forces between polymeric chains [67,68]. These findings align with those in the literature, which report that the microhardness of dental composites is higher before water storage than after [69,70]. However, the effect of water on surface microhardness is material-dependent, and not all dental composites will experience a decrease in hardness after short-term water exposure [71]. It has been widely reported that the polymerization of resin composites continues for up to 24 h following light curing [72,73,74]. Therefore, microhardness was measured one week after fabrication.

In the second phase of the study, creep measurements were conducted. Dental resin composites are polymer materials with time-dependent mechanical properties [53,75]. The viscoelastic properties of resin composite materials represent a critical aspect of their mechanical performance [76,77] and have been extensively investigated and highlighted in numerous studies [78,79,80,81]. High loading forces during mastication can lead to the failure of composite restorations, making viscoelastic properties, such as creep strain, essential to consider. In clinical situations, strain recovery occurs during unloading phases [82]. According to Baroudi et al., composites with high creep deformation exhibit poor resistance to mechanical stress, which may adversely affect the long-term clinical durability of restorations [83]. To our knowledge, the creep behavior of flowable SFRC has not been previously tested. In the present study, the bulk application of flowable SFRC (Group 3) demonstrated significantly higher modulus (E1 and E2) and viscosity (*η*) values, as well as better resistance to creep compared to the bulk PFC (SDR, Group 4) (*p* < 0.05), confirming its suitability for use without surface coverage. The incorporation of microfibers in the composite material enhances its modulus and improves creep and fatigue resistance [17,84]. Creep, as a viscoelastic characteristic, is primarily influenced by the filler content, with higher filler loading generally resulting in decreased creep in these materials [85,86]. However, the extent of creep in resin-based materials also depends on the type and quantity of the resin component, as well as the changes induced by thermal effects [77,87]. According to Watts, creep resistance reflects the viscoelastic stability of a material and its ability to resist catastrophic failure under loading [81]. This aligns with findings on modern SFRC materials, particularly their ability to transform irreparable failures into repairable ones [30,65,88]. In the study conducted by Molnár et al., a fracturegraphy analysis revealed that the primary crack originated from the occlusal surface of the restoration, propagated downward, and extended through the various layers of the restoration and tooth structure [64].

Several studies have demonstrated that the SFRC substructure supports composite restorations and functions as a crack prevention layer [89,90,91]. According to Garoushi and colleagues, the thickness of the SFRC substructure plays a critical role as it influences the failure mode and the crack-arresting mechanism [92]. Moreover, the thickness of the veneering composite also significantly affects the restoration’s performance. In this study, the dimensions of deep Class I restorations utilizing flowable SFRC without conventional composite coverage (Groups 2 and 3) were simulated and evaluated for static nanoindentation, nanoindentation creep, and water uptake. The authors consider this approach not only novel, but also a strength of the study, given the growing body of literature on flowable SFRC restorations without conventional composite coverage, which has demonstrated remarkable mechanical performance [30,32,93,94]. Consequently, it is essential to investigate all surface-related parameters, including bacterial adhesion, gingival irritation, and aesthetic parameters associated with this restorative option.

In our study, the water uptake of the tested direct resin composite specimens was also evaluated. Bulk SFRC (Group 3) demonstrated significantly lower water absorption compared to the other groups (*p* < 0.001), which aligns with findings from previous research in the literature [24]. The water uptake of resin composites is primarily influenced by the hydrophilicity and cross-linking of the network structure. Additionally, porosity, as well as the nature of the filler and the filler/fiber–matrix interface, contribute to the extent of water uptake during the exposure period. Interestingly, the bulk application of SFRC showed less water uptake than the layering technique, which may indicate that the water uptake process is facilitated by the diffusion of water molecules into voids or porosity at the interfaces between layers [95]. This can lead to hydrolytic degradation and undesirable anisotropic behavior, potentially compromising the uniformity and mechanical integrity of the restoration [96].

Resin composites are widely used in restorative procedures due to their aesthetic properties and mechanical performance. However, their compatibility with biological tissues remains a critical consideration, particularly in cases where materials may be in close proximity to soft tissues or exposed to pulpal environments. The biocompatibility of these materials is influenced by factors such as the type and concentration of monomers, the degree of polymerization, and the release of potentially cytotoxic substances during and after curing [97]. Inadequate curing or the presence of residual monomers may lead to adverse cellular responses, including inflammation, cytotoxicity, or reduced cell viability. Therefore, evaluating the tissue response to different composite formulations is essential to ensure their clinical safety and effectiveness.

According to Attik et al., flowable SFRC (everX Flow) exhibited a less deleterious effect on primary gingival cell viability compared to a bulk-fill particulate filler composite (SDR), particularly on day 3 [57]. This trend persisted through to day 5, with a noted enhancement in cellular metabolic activity in the presence of SFRC. These findings support the favorable biological response of SFRCs, further endorsing their use in direct restorative dental applications.

The orientation and distribution of fibers in SFRCs play a critical role in determining their mechanical properties and resistance to water sorption. Randomly oriented fibers can provide isotropic reinforcement, offering uniform mechanical strength in all directions, which is particularly advantageous for complex stress conditions. However, a more aligned fiber orientation tends to improve mechanical performance along the primary load-bearing direction, enhancing properties such as flexural strength and fracture toughness [98]. In contrast, uneven or clustered fiber distribution can lead to localized stress concentrations and reduced structural integrity [84]. Moreover, fiber orientation and packing density influence the composite’s porosity and the interfacial bonding between the fibers and the resin matrix, which are factors that directly affect water sorption. Poorly distributed fibers may create microvoids that facilitate water uptake, potentially accelerating hydrolytic degradation. Thus, optimizing both fiber orientation and distribution is essential for maximizing mechanical performance and minimizing water-related deterioration in short fiber composites.

This study has some limitations. First, the nanoindentation test is highly sensitive and has a narrow measurement range, which could lead to variable results depending on whether the indentation occurs over the fiber surface or the matrix. However, 19 indents were conducted per specimen to ensure reliable and representative measurements. Second, solubility was not measured, which would have provided a broader understanding of the material’s degradation behavior. Third, only a limited number of materials were tested, and the findings may not fully represent the wide range of commercially available fiber-reinforced and bulk-fill composites. Different products can vary significantly in terms of fiber/filler type, orientation, distribution, and resin composition, all of which may influence mechanical performance and degradation characteristics. Future studies should aim to include a broader selection of commercial materials to better assess the generalizability of the results.

## 5. Conclusions

This study investigated the nanomechanical properties and water uptake of a flowable SFRC using various restorative techniques. The results demonstrated that bulk-applied SFRC exhibited superior mechanical behavior and significantly lower water absorption compared to conventional and bulk-fill PFCs. These findings support its use as a standalone restorative material without the need for covering.

This study also highlights the importance of the application technique, as layering versus bulk placement influenced material behavior. Among all groups, bulk-applied SFRC showed the most favorable combination of mechanical strength and water resistance, suggesting it may offer both simplified handling and reliable performance in clinical practice.

In conclusion, flowable SFRC applied in bulk without surface coverage presents a promising alternative to conventional composites. Its favorable nanomechanical properties and reduced susceptibility to water-induced degradation support its potential as an efficient and durable restorative material. These findings contribute to the growing body of evidence supporting SFRCs as standalone direct restorative materials and provide a strong foundation for future clinical investigations.

## Figures and Tables

**Table 1:** Composition of the investigated resin composites.

Material (Code)	Manufacturer	Organic Matrix	Fillers (wt.%)
everX Flow Bulk Shade	GC Europe, Leuven, Belgium	Bis-EMA, TEGDMA, UDMA	Short glass fiber (200–300 μm & Ø6 μm), barium glass. 70%
SDR flow+, Bulk Fill (SDR)	Dentsply, DeTrey, Konstanz, Germany	modified UDMA, EBPADMA, TEGDMA	Barium-alumino-fluoroborosilicate glass, strontium alumino-fluoro-silcate glass. 68%
G-aenial Posterior A2 (PFC)	GC Europe, Leuven, Belgium	UDMA, dimethacrylate co-monomers	Fluoroaluminosilicate glass, fumed silica, pre-polymerized fillers. 77%

UDMA: urethane dimethacrylate; TEGDMA: triethylene glycol dimethacrylate; EBPADMA: ethoxylated bisphenol A dimethacrylate; Bis-EMA: bisphenol A ethoxylated dimethacrylate.

**Table 2:** Description of groups used in this study.

Group	Material	Application Technique
1 (Control)	PFC	Layered (2–2–1 mm)
2	SFRC	Layered (2–2–1 mm)
3	SFRC	Bulk
4	Bulk-fill PFC	Bulk
5	SFRC + PFC	Layered (2–2–1 mm)

## Data Availability

The original contributions presented in this study are included in the article.
